# Reduction of the pulse duration of the ultrafast laser pulses of the Two-Photon Laser Scanning Microscopy (2PLSM)

**DOI:** 10.1186/1756-0500-1-39

**Published:** 2008-07-09

**Authors:** Ali Hussain Reshak

**Affiliations:** 1 Institute of Physical Biology-South Bohemia University, Institute of System Biology and Ecology-Academy of Sciences – Nove Hrady 37333, Czech Republic

## Abstract

**Background:**

We provide an update of our two-photon laser scanning microscope by compressing or reducing the broadening of the pulse width of ultrafast laser pulses for dispersion precompensation, to enable the pulses to penetrate deeply inside the sample.

**Findings:**

The broadening comes as the pulses pass through the optical elements. We enhanced and modified the quality and the sharpness of images by enhancing the resolution using special polarizer namely Glan Laser polarizer GL10. This polarizer consists of two prisms separated by air space. This air separation between the two prisms uses to delay the red wavelength when the light leaves the first prism to the air then to second prism. We note a considerable enhancing with using the GL polarizer, and we can see the details of the leaf structure in early stages when we trying to get focus through z-stacks of images in comparison to exactly the same measurements without using GL polarizer. Hence, with this modification we able to reduce the time of exposure the sample to the laser radiation thereby we will reduce the probability of photobleaching and phototoxicity. When the pulse width reduced, the average power of the laser pulses maintained at a constant level. Significant enhancement is found between the two kinds of images of the Two-Photon Excitation Fluorescence (TPEF).

**Conclusion:**

In summary reduction the laser pulse width allowed to collect more diffraction orders which will used to form the images. The more diffraction orders the higher resolution images.

## 1. Introduction

Non-linear optical techniques have been exploited to develop a new generation of optical microscopes with unprecedented capabilities. These new capabilities include the ability to use near-infrared (IR) light to induce absorption, and hence fluorescence, from fluorophores that absorb in the ultraviolet wavelength region. Other capabilities of non-linear microscopes include improved spatial and temporal resolution without the use of pinholes or slits for spatial filtering, improved signal strength, deeper penetration into thick, highly scattering tissues, and confinement of photobleaching to the focal volume [1]. Two-photon excitation offers major advantages when working in the thick tissue, such as brain slices or developing embryos, due to the dramatically reduced effects of light scattering. This is partly because the longer red and near-IR wavelengths used for two photon illumination penetrate deeper into biological tissue with less absorption and scattering. However, the main advantage comes from the non-linear excitation. The requirement for two coincident (or near coincident) photons to achieve excitation of the fluorophore means that only focused light reaches the required intensities and that scattered light does not cause excitation of the fluorophore.

The introduction of two-photon excitation [1] to life sciences has opened novel experimental territories [2]. Two-Photon Laser Scanning Microscopy is a fluorescence imaging technique that allows imaging living tissue up to depth of one millimeter. It is a special variant of the multiphoton fluorescence microscopy. Two-Photon excitation may in some cases be a viable alternative to confocal microscopy due to its deeper tissue pentration and reduced phototoxicity [1]. It is employs a concept first described by Maria Goppert-Mayer (b. 1906) in her 1931 doctoral dissertation [3].

The use of infrared light to excite fluorophores in light-scattering tissue has added benefits [4]. Longer wavelengths are scattered to a lesser degree than shorter ones, which is a benefit to high-resolution imaging. In addition, these lower-energy photons are less likely to cause damage outside of the focal volume.

Ultrashort pulse systems have a unique set of characteristics caused by the high peak powers and temporal conditions, which must be considered in their design. The envelope of an ultrashort pulse contains a large number of frequencies; hence, such pulses have very large bandwidths. This bandwidth sets the limit for the shortest pulse duration with the relationship

(1) Δ*τ*_*p *_Δ*ν *= *X*

where Δ*τ*_*p *_is the temporal full-width half-maximum of the pulse and Δ*ν *is the spectral bandwidth. The value *X *will be a minimum when the pulse is said to be fourier-transform-limited, at which point the spectral bandwidth is unable to support shorter pulse durations. In optical materials, the refractive index is frequency dependent. This dependence can be calculated for a given material using a Sellmeier equation, typically of the form:

(2) $$n^{2}\left(\lambda \right)=1+\frac{B_{1}\lambda^{2}}{\lambda^{2}-C_{1}}+\frac{B_{2}\lambda^{2}}{\lambda^{2}-C_{2}}+\frac{B_{3}\lambda^{2}}{\lambda^{2}-C_{3}}$$

where *B*_1,2,3 _and *C*_1,2,3 _are the Sellmeier constants derived from experimental data. Equation 2 is valid over a range of wavelengths dependent on the material it describes. Hence, dispersion arises from the fact that light of different wavelengths travels through the material at different speeds. In a normally dispersive material blue light travels more slowly and is refracted more than red light.

Frequency components within a pulse will travel with a unique phase velocity of *ν*_*φ *_= *c*/*n*(*λ*) through a dispersive medium. Pulse broadening occurs when the faster components extend the leading edge of the pulse envelope, while the slower components retard the trailing edge. The instantaneous velocity of this pulse envelope is called the group velocity, *v*_*g*_. Group velocity dispersion (GVD) is often responsible for producing linear phase changes or chirp across the pulse.

**Figure 1:**
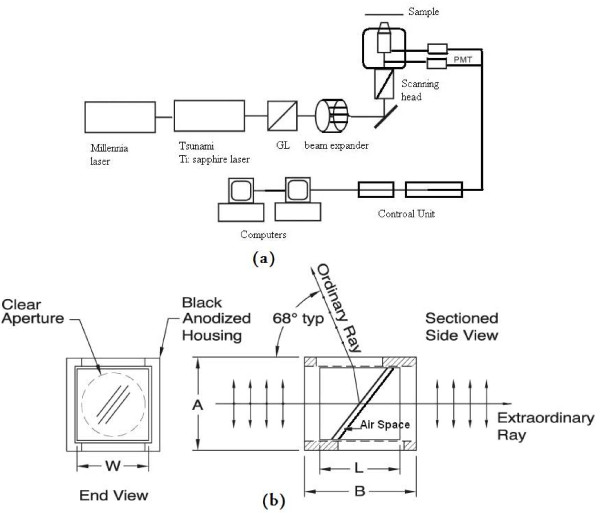
(a) Experimental setup. PMT: photomultiplier; GL: Glan Laser polarizer is used to make the incident laser purely polarized. (b) Diagram of GL10 Glan Laser Polarizer.

The majority of pulse broadening in ultrashort pulse lasers is caused by the positive group-velocity dispersion of the gain medium. Other intracavity elements such as prisms will also contribute positive dispersion. To obtain the shortest possible pulses from the laser cavity the overall GVD has to be near zero. A practical method for doing this is to introduce pairs of prisms into the cavity, as described by Fork et al. in 1984 [5]. This is known as dispersion compensation. The prism material will itself contribute positive dispersion, but it is possible to configure the prism pairs so that the overall contribution is negative (see Fig. 1). Kang et al. [6] generate negative group velocity dispersion by a single prism and wedge mirror in femtosecond lasers. They discuss that both theoretical analyses and the experimental results show that the GVD is directly proportional to the distance between the prism and the Ti:sapphire crystal. Also they prove that the amount of GVD generated by this method approach that generated by a pair of prisms. Andreas et al. [7] used dielectric mirrors for group-delay dispersion control of s laser cavity free from the problems of cubic dispersion, asymmetric spectra and increased sensitivity of pulse width to cavity and prism alignment. By use of Kerr-lens mode locked Ti:sapphire laser without any intracavity prisms for generating highly stable optical pulses as short as 11 fs. Recently Zeng et al. [8,9] introduce a single prism before the two dimensional acousto-optical deflector (AOD) to allows simultaneous compensation of spatial and temporal dispersion for two-dimensional scanning.

A prism sequence that provides a way of introducing GVD that is low loss and is adjustable through both positive and negative values. The prisms are cut and orientated so that the rays are incident at minimum deviation and Brewster's angle, to minimize losses. The total dispersion of the prism sequence is calculated as

(3) $$D=\left[\frac{\lambda }{cL}\right]\frac{d^{2}P}{d\lambda^{2}}$$

where *L *is the physical length of the light path, P is the optical path length. The derivative d^2^*P*/d*λ*^2 ^is a function of the angular divergence *α*, the refractive index of the prism material and the apex separation, *l*, of the prisms. It can be shown that for sufficiently large values of *l *the overall dispersion becomes negative. Therefore, by changing the prism positions it is possible to vary the total dispersion of the cavity from positive to negative. The geometric configuration of the prism pairs can only shorten Ti:sapphire laser pulses to the sub-100 fs regime before which third order dispersion becomes a limiting factor. In order to achieve shorter pulse durations it is necessary to choose a prism glass that has a combined geometric and material contribution which compensates the third order dispersion of the gain medium [10].

Another way of controlling group velocity dispersion, without the need of introducing intracavity glass, is by using chirped dielectric mirrors as the source of broadband negative GVD [7,11]. Such mirrors have facilitated the generation of sub-5 fs laser pulses [12]. The general formula of group delay dispersion (GDD) as obtained for a general arrangement of the prisms can be written as [13];

(4) $$GDD=\frac{\lambda^{3}}{2\pi^{2}c^{2}}\frac{d^{2}P}{d\lambda^{2}}$$

## 2. Materials

The experimental arrangement is shown in Figure 1. We used inverted i-mic 2 microscope, Till-Photonics, Grafelfing, Germany [14], equipped with Ti:sapphire femtosecond laser with a tuning range of 690 nm to 820 nm. The lasers we currently using are Tsunami 3941-M3B pumped by Millennia-V, 5W solid-state pump laser (Spectra-Physics). Ti:sapphire femtosecond laser was used to generate flashes 760 nm, 20 mW, and 80 fs pulse width for excitation. Beam expander was used to fill the back aperture of the objective. The excitation light was directed onto a pair of galvanometer XY scanner (Yauns-Till-Photonics). The scanned excitation light was focused onto the specimen through the microscope objective (Olympus objectives type uplanFLN 10×/0.30 or uplanApo/IR 60×/1.20 water immersion) in order to scan the specimen in the x-y direction at the focal plane. The stage of the microscope is driven by a computer controlled motor to take the specimen to different z positions following each x-y scan. Scanning mirrors are metal coated (silver) and have a good thermal resistance [15]. Fluorescence emitted at the focal plane was directed via a dichroic beam splitter (740DCXR). Dichroic beam splitter separated the emissions from the excitation light. The fluorescence signals are separated from each other by two dichroic beam splitters (upper channel and lower channel) and directed to individual photomultipliers (PMT) (Hamamatsu R6357). The desired emission filter placed into the beam path before the photomultiplier. IR beam block filter is places in front each PMT to ensure that illumination light was filtered out and only TPEF signals were recorded. The signals from the photomultipliers are reconstructed by computer hardware into image. Images were obtained in stacks scanning along the z-axis with 0.5 μm steps. The microscopy is controlled via a standard high-end Pentium PC and linked to the electronic control system via an ultrafast interface.

We enhanced and modified the quality and the sharpness of images by enhancing the resolution using special polarizer namely Glan Laser polarizer GL10 from THORLABS (see Fig. 1b and Table 1). The Glan Laser Calcite Polarizer is manufactured from select portions of the calcite crystal, making it an appropriate choice for high energy laser applications. They provide extremely pure linear polarization (100,000:1). Two polished side ports are provided to allow bi-directional use of the polarizer. The input and output faces are polished to a laser quality 20-10 scratch-dig surface finish to minimize scattering of the transmitted P polarization component of the laser beam or light field. The S polarization component is reflected through a 68° angle and exits the polarizer through one of the two side ports, which are pad polished. This polarizer consists of two prisms separated by air space. This air separation (air space) between the two prisms uses to delay the red wavelength when the light leaves the first prism to the air then to second prism. This polarizer acts exactly the same with the prism pair to reduce the pulse duration. The polarizer was placed directly after Brewster widow before the beam expander (see Fig. 1a). In the current measurements we used *duckweed Lemna minuta*. *Lemna *is a genus of free-floating aquatic plants from the duckweed family. These rapidly-growing plants have found uses as a model system for studies in basic plant biology, in ecotoxicology, in production of biopharmaceuticals, and as a source of animal feeds for agriculture and aquaculture. Plant body flattened or with inflated (gibbous) ventral side, generally oval-oblong in outline with 1–3 (5) veins, 2–5 mm long (longer in L. trisulca), pale (transparent) green or dark green, often suffused with reddish anthocyanin; floating solitary are attached in clusters of 2–8 (or more) by a short stipe (or elongate stipe in L. trisulca); dorsal surface often with row of papules along midline; budding pouches 2, on either side (lateral) of basal end; daughter plants produced in budding pouches (turions also produced in pouches of L. turionifera); needlelike (raphide) crystals of calcium oxalate throughout parenchyma; solitary root with basal root sheath near attachment on ventral side, thicker fronds often with deep root furrow (groove); one bisexual flower produced within membranous spathe (utricular scale) inside each lateral budding pouch, consisting of a single pistil flanked by 2 stamens (some authorities consider this to be an inflorescence with 3 unisexual flowers); anthers bilocular and transversely dehiscent; ovary unilocular with 1 amphitropous or orthotropous ovule (or 2–4 anatropous ovules); utricle globular and slightly compressed (sometimes winged), bearing 1-several longitudinally-ribbed seeds (rarely smooth), some species with transverse striations between ribs, seeds with distinct operculum; 13 spp.

**Table 1:** specification of Glan Laser polarizer GL10

**Item Number**	GL10
**Extinction Ratio**	100,000:1
**Material**	Laser Quality Natural Calcite (low scatter)
**Design**	High Laser Damage Threshold Air-Spaced Design
**Damage Threshold**	500 W/cm^2 ^of CW Power 500 MW/cm^2^(10 ns Pulse 1.06 μm)
**Wavefront Distortion**	≤ λ/4 Over Clear Aperture
**Scratch/Dig (Input and Output Faces**	20/10
**Scratch/Dig (Side Ports)**	80/50
**Transmission Range**	0.35 – 2.3 μm
**Aperture**	10 mm × 10 mm
**Prism Dimensions (W × L)**	12 mm × 13.7 mm
**-A Coating Wavelength Range**	300–600 nm
**-B Coating Wavelength Range**	600–900 nm
**-C Coating Wavelength Range**	1250–1620 nm
**V-Coat Wavelength**	1.06 μm

This species have been studied extensively for use in phytotoxicity tests. Genetic variability in responses to toxicants can occur in *Lemna*, and there are insufficient data to recommend a specific clone for testing.

## 3. Results and Discussions

Deeply penetration in side the sample needs to increase the average power of the laser pulse. But due to the damage limit due to sample heating, we are bounded with 1–5 mW which is usually used for multiphoton imaging at conventional scan speeds. In fact only 100 mW is really needed from the laser at most wavelengths, because the loss of most laser power in the optical equipments, finally we will get only 1/3 (for our microscope) of the laser average power at the sample. The other factor is *P*_*Peak *_∝ 1/*τ*;, where *τ*, is the pulse duration. From this expression one can understand that *P*_*Peak *_depends on how short a pulse can be delivered to the sample. The benefit of scaling up peak power by reducing pulse width will avoid sample damage due to heating. The pulse duration *τ*_*sample *_is directly proportional to the group velocity dispersion (GVD), *τ*_*sample *_∝ GVD.

The resolution of the images is related to the numerical aperture number, the later is directly related to the cone of light from the specimen at its vertex which is brought into the lens. In general when the light hits an object, it diffracts. A single beam of light will be split into several different diffraction orders bent at increasing angles from the original impinging beam. Resulting in a large central disk of light surrounded by a series of thin concentric circles of light of decreasing brightness the further away from the center they are. The same will happen when light hits a microscopic specimen, the diffraction orders spread out. The bigger cone of light brought into the objective, can collect more diffraction orders with more information. The higher numerical aperture will brought bigger cone of light. Therefore the higher numerical aperture objective gives better resolution. The second advantage of using a higher numerical aperture is that since more orders of diffraction form the object are brought into the lens, more light generally is brought into higher numerical aperture lens producing brighter images.

From above we can conclude that compressing the pulse width of the laser beam which hits the object bends more light into the objective capturing more orders of diffraction from the object, keeping in mind that finer details or more closely spaced objects will give much higher angles of diffraction than will larger objects with less fine details. With this reduction of the laser pulse width we will allow the low numerical aperture (N.A.) objective to collect more diffraction orders may be in the same efficiency of the higher N.A. objective used without reduction the laser pulse width. In summary reduction the laser pulse width allowed to collect more diffraction orders which will used to form the images. The more diffraction orders the higher resolution images.

**Figure 2:**
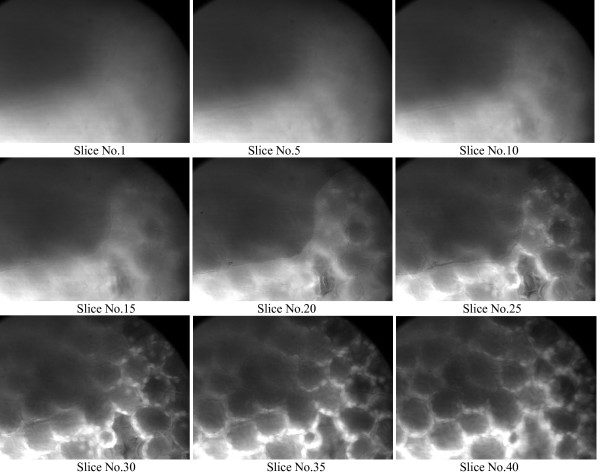
The structure of the leaf using normal 2PLSM.

**Figure 3:**
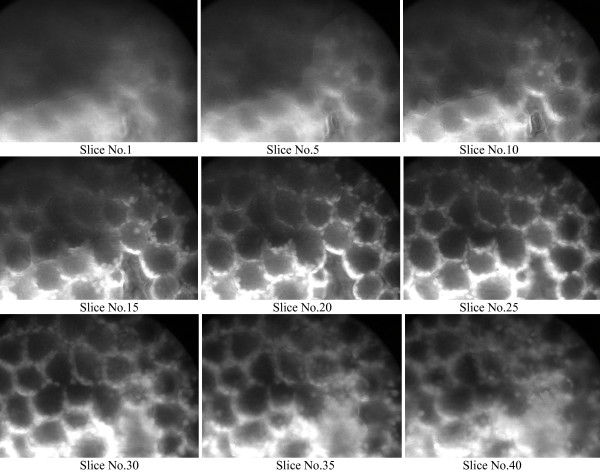
The structure of the leaf using modified 2PLSM.

Figure 2, 3, and the additional files 1 and 2, show the leaf structure of *duckweed Lemna minuta *excited by laser beam without and with using GL polarizer. In Figure 3 and the additional file 2, we note a considerable enhancing with using the GL polarizer, and we can see the details of the leaf structure in early stages when we trying to get focus through z-stacks of images going though the leaf from the dorsal surface to ventral surface in comparison to the exactly the same measurements without using GL polarizer. In this case we can see the structure of the leaf (which we saw it in the first slices with using GL polarizer) approximately after 15 slices, i.e we have to go deeply inside the sample about 7.5 μm (separation between the slices is 0.5 μm) before using the GL polarizer. Hence, with this modification we able to reduce the time of exposure the sample to the laser radiation thereby we will reduce the probability of photobleaching and phototoxicity. If we look at Figure 2 and the additional file 1, we note that without using the GL polarizer the images will be blurred and we need more focusing to get clearer images resulting to exposure the sample more time to the laser radiation. This improvement over the normal 2PLSM will enable us to get quick focus through the sample with high resolution and give us more opportunity to go deeply in side the tissue to see clearer details structures with less damage to the living cells. We provided two movies (additional files 1 and 2) which show the leaf structures with and without using GL polarizer. These movies clearly show the resolution enhancement with GL polarizer.

## 4. Conclusion

In this work we provide a substantil update to our two photon laser scanning microscopy. The major improvements are enabling higher resolution with deeply penetration in side the living cell without photobleching and phtotoxicity. We achieved this improvement by using Glan Laser polarizer GL10 with air space. The air space introduces negative dispersion by delaying the longer wavelength component. With this polarizer we can gain very clear and sharp images. We can get the same clearness and sharpness images without using the GL polarizer but have to go deeply by around 7.5 μm in side the sample. So with using the GL polarizer we can easily focus in side the sample with less time and keeping the sample healthy.

## Supplementary Material

Additional File 1 The structures of the leaf before the enhancement. The movie represents the structure of *duckweed Lemna minuta *leaf before the modification of our two photon laser scanning microscope. Click here for file

Additional File 2 The structures of the leaf after the enhancement. The movie represents the structure of *duckweed Lemna minuta *leaf after the modification of our two photon laser scanning microscope. Click here for file
